# Functional assessment of missense variants of uncertain significance in the cancer susceptibility gene *PALB2*

**DOI:** 10.1038/s41523-022-00454-6

**Published:** 2022-07-19

**Authors:** Shijie Wu, Lina Qi, Huihui Chen, Kun Zhang, Jiapan He, Xianan Guo, Lu Shen, Yunxiang Zhou, Xi Zhong, Shu Zheng, Jiaojiao Zhou, Yiding Chen

**Affiliations:** 1 Department of Breast Surgery and Oncology, Key Laboratory of Cancer Prevention and Intervention, Ministry of Education, the Second Affiliated Hospital, Zhejiang University School of Medicine, Hangzhou, 310009 Zhejiang China; 2 Department of Medical Oncology, Key Laboratory of Cancer Prevention and Intervention, Ministry of Education, the Second Affiliated Hospital, Zhejiang University School of Medicine, Hangzhou, 310009 Zhejiang China; 3 Cancer Center, Zhejiang University, Hangzhou, 310009 Zhejiang China

**Keywords:** Breast cancer, Cancer genetics

## Abstract

Germline *PALB2* pathogenic variants are associated with an increased lifetime risk for breast, pancreatic, and ovarian cancer. However, the interpretation of the pathogenicity of numerous *PALB2* missense variants of uncertain significance (VUSs) identified in germline genetic testing remains a challenge. Here we selected ten potentially pathogenic *PALB2* VUSs identified in 2279 Chinese patients with breast cancer and evaluated their impacts on PALB2 function by systematic functional assays. We showed that three *PALB2* VUSs p.K16M [c.47 A > T], p.L24F [c.72 G > C], and p.L35F [c.103 C > T] in the coiled-coil domain impaired PALB2-mediated homologous recombination. The p.L24F and p.L35F variants partially disrupted BRCA1-PALB2 interactions, reduced RAD51 foci formation in response to DNA damage, abrogated ionizing radiation-induced G2/M checkpoint maintenance, and conferred increased sensitivity to olaparib and cisplatin. The p.K16M variant presented mild effects on BRCA1-PALB2 interactions and RAD51 foci formation. Altogether, we identify two novel *PALB2* VUSs, p.L24F and p.L35F, that compromise PALB2 function and may increase cancer risk. These two variants display marked olaparib and cisplatin sensitivity and may help predict response to targeted therapy in the clinical treatment of patients with these variants.

## Introduction

Partner and localizer of BRCA2 (PALB2) is crucial for homologous recombination (HR) repair in response to DNA double-strand breaks (DSBs)^1^. PALB2 serves as a tumor suppressor and contributes to the maintenance of genome integrity. Biallelic pathogenic variants in *PALB2* are known to result in a subtype of Fanconi anemia (FA-N), whereas monoallelic pathogenic variants increase the risk of breast, pancreatic, and ovarian cancer^2–4^. Notably, *PALB2* protein-truncating variants are associated with a 30–60% increased risk of breast cancer^4–7^. Some studies have also indicated that *PALB2*-mutated breast cancer is closely correlated with aggressive clinicopathological features, including triple-negative phenotype, advanced disease stage, high Ki67 levels, and poor prognosis^8,9^.

*PALB2* encodes an 1186-amino acid protein with several functional domains, including a coiled-coil domain, a chromatin-association motif (ChAM), a MRG15-binding domain, and a WD40 domain^10^. In response to DNA DSBs induced by genotoxic agents, PALB2 mainly serves as a bridging molecule that interacts with BRCA1 and BRCA2 using the N-terminal coiled-coil domain and C-terminal WD40 domain, respectively^1,11,12^. The combined BRCA1-PALB2-BRCA2 complex then recruits RAD51 and stimulates RAD51-mediated HR, ultimately completing the high-fidelity repair of DSBs^10,13–15^. Besides the vital role in HR repair, PALB2 also acts as a versatile player in the regulation of biological processes, including cell-cycle checkpoint control^16,17^, cellular redox homeostasis regulation^18^, protection of actively transcribed genes^19^, and recovery of stalled DNA replication forks^20,21^. These findings shed light on the importance of PALB2 in maintaining genome stability and explained why PALB2 deficiency often leads to genome instability syndromes, such as cancer predisposition.

Based on the genetic testing of cancer patients and their relatives in recent years, a large number of *PALB2* variants have been discovered. However, a major obstacle is that numerous identified variants are missense variants of uncertain significance (VUSs). The effects of these VUSs on HR and their association with increased breast cancer risks are often unknown, posing a challenge for genetic counselling and clinical variant classification. Recently, Foo et al. reported the first likely pathogenic *PALB2* VUS p.L35P [c.104 T > C] using systematic functional assays. The p.L35P variant abolishes the BRCA1-PALB2 interaction and completely disrupts the HR function of PALB2, resulting in hypersensitivity to cisplatin and a poly (ADP-ribose) polymerase (PARP) inhibitor (PARPi)^22^. Three recent studies further provided a comprehensive analysis of *PALB2* VUSs and identified major regions for VUSs affecting PALB2 function^23–25^. In the N-terminal coiled-coil domain, pathogenic variants mainly disrupt BRCA1-PALB2 binding and lead to compromised HR function. In the C-terminal WD40 domain, HR deficiency of PALB2 is associated with reduced PALB2-BRCA2 interaction, PALB2 protein instability, and cytoplasmic translocation of PALB2. However, more than 92% of *PALB2* missense variants are still defined as VUSs^6^. In this study, we evaluated the impact of breast cancer patient-derived VUSs on PALB2 function and aimed to identify pathogenic *PALB2* missense variants that may increase cancer risk.

## Results

### Selection of patient-derived *PALB2* missense variants for functional analysis

**Fig. 1:**
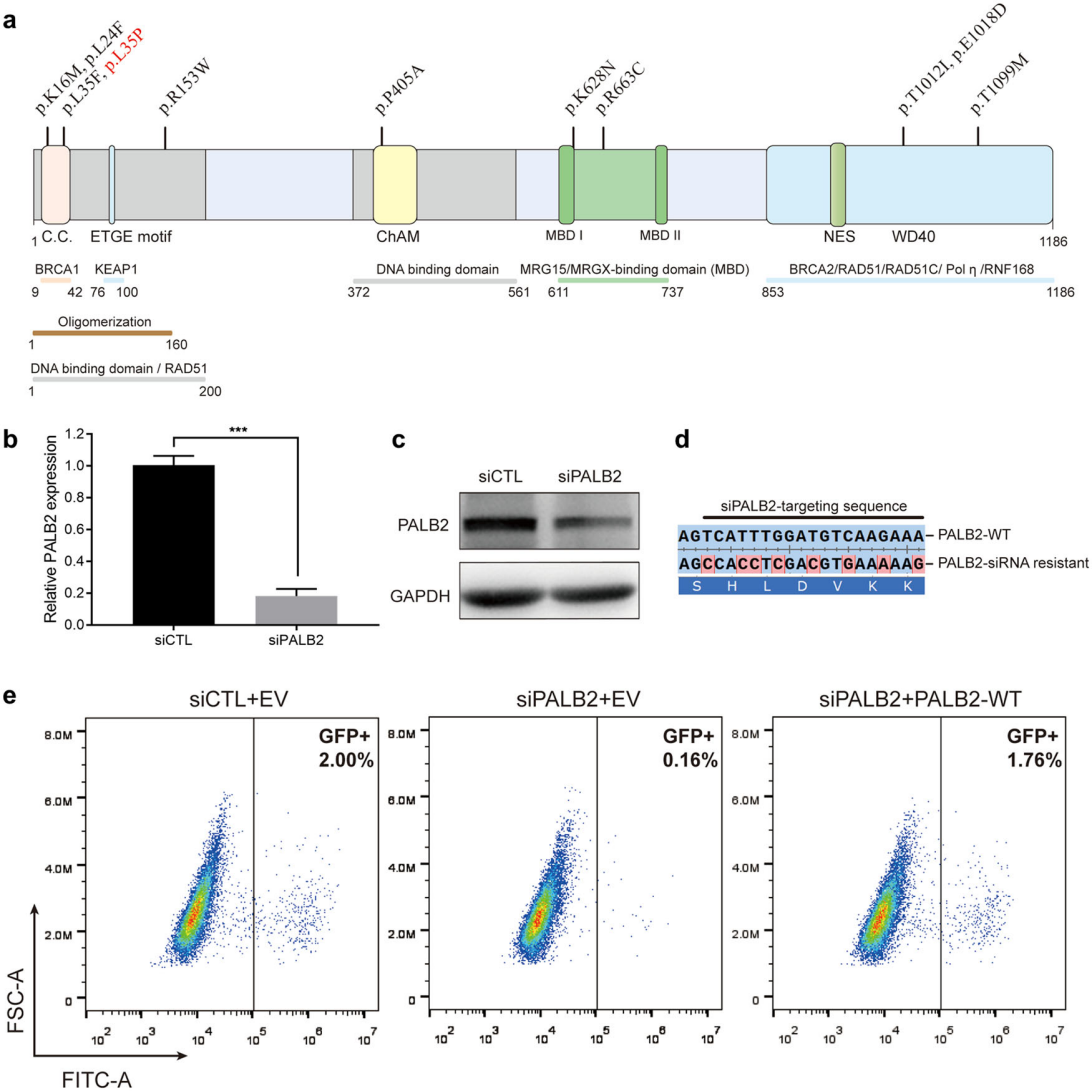
Overview of *PALB2* VUSs examined in our study and validation of the U2OS/DR-GFP homologous recombination (HR) reporter system. **a** Schematic representation of the PALB2 protein and the position of the previously identified *PALB2* VUSs. The p.L35P pathogenic variant was also included and highlighted in red. C.C., coiled-coil domain; ChAM, chromatin-association motif; WD40, WD40-repeats; NES, nuclear export sequence. **b**, **c** siRNA-mediated knockdown efficiency of endogenous PALB2 in U2OS/DR-GFP cells was evaluated by quantitative PCR and western blotting. Data represent the mean percentage (± SEM) of PALB2 mRNA relative to siCTL from three independent experiments. Statistical significance was analyzed by Student’s *t*-test. ***P < 0.001. **d** Schematic representation of the siRNA-resistant *PALB2* cDNA containing eight silent base changes. **e** Representative plots for the HR activity of PALB2. The HR activity of U2OS/DR-GFP cells was first deprived after siRNA knockdown and then rescued with the siRNA-resistant PALB2-WT construct by transient transfection. GFP-positive cells were quantified by flow cytometry. EV Empty vector, WT Wild type.

Our previous study identified 31 *PALB2* missense variants in 2279 Chinese patients with breast cancer^26^. These *PALB2* variants were assessed by SIFT^27^, Align GVGD^28^, and Polyphen-2^29^ in silico. Among them, 12 missense variants were classified as potentially pathogenic by at least two in silico algorithms^26^. The variants in the functional domains, including the coiled-coil domain (p.K16M, p.L24F, and p.L35F), DNA-binding domain (p.R153W), ChAM (p.P405A), MRG15-binding domain (p.K628N and p.R663C), and WD40 domain (p.T1012I, p.E1018D, and p.T1099M), may disturb the function of PALB2, resulting in the cancer predisposition. Thus, we selected the above 10 potentially pathogenic *PALB2* variants for functional analysis based on the amino acid substitution position (Fig. 1a). Details relative to the selected *PALB2* variants were presented in Table 1. In addition, we included the p.L35P [c.104 T > C] variant as a negative control, for which pathogenicity has recently been confirmed^22^.

**Table 1:** The *PALB2* variants with in silico assessment by prediction tools.

Location	DNA change	Protein change	Number of heterozygotes in all BCa cases (%)	Number of heterozygotes in Familial BCa cases (%)	Number of heterozygotes in Sporadic BCa cases (%)	SIFT prediction	Align GVGD prediction	Polyphen-2 prediction
Exon 1	c.47 A > T	p.K16M	1 (0.04)	-	1 (0.05)	Damaging (0)	Class C0	Probably Damaging (1)
Exon 2	c.72 G > C	p.L24F	1 (0.04)	1 (0.33)	-	Damaging (0)	Class C15	Probably Damaging (1)
Exon 2	c.103 C > T	p.L35F	1 (0.04)	1 (0.33)	-	Damaging (0)	Class C15	Probably Damaging (1)
Exon 4	c.457 A > T	p.R153W	1 (0.04)	-	1 (0.05)	Damaging (0.01)	Class C0	Probably Damaging (0.997)
Exon 4	c.1213 C > G	p.P405A	13 (0.57)	3(0.98)	10 (0.51)	Damaging (0)	Class C25	Probably Damaging (1)
Exon 5	c.1884G > T	p.K628N	1 (0.04)	-	1 (0.05)	Damaging (0.02)	Class C0	Probably Damaging (0.998)
Exon 5	c.1987C > T	p.R663C	1 (0.04)	-	1 (0.05)	Damaging (0)	Class C15	Probably Damaging (0.995)
Exon 10	c.3035 C > T	p.T1012I	4 (0.18)	1 (0.33)	3 (0.15)	Damaging (0)	Class C15	Probably Damaging (1)
Exon 10	c.3054 G > C	p.E1018D	33 (1.45)	4 (1.30)	29 (1.47)	Damaging (0.02)	Class C0	Probably Damaging (0.998)
Exon 12	c.3296 C > T	p.T1099M	2 (0.08)	-	2 (0.10)	Damaging (0)	Class C15	Probably Damaging (1)

*BCa* Breast cancer.

### HR activity of *PALB2* variants

**Fig. 2:**
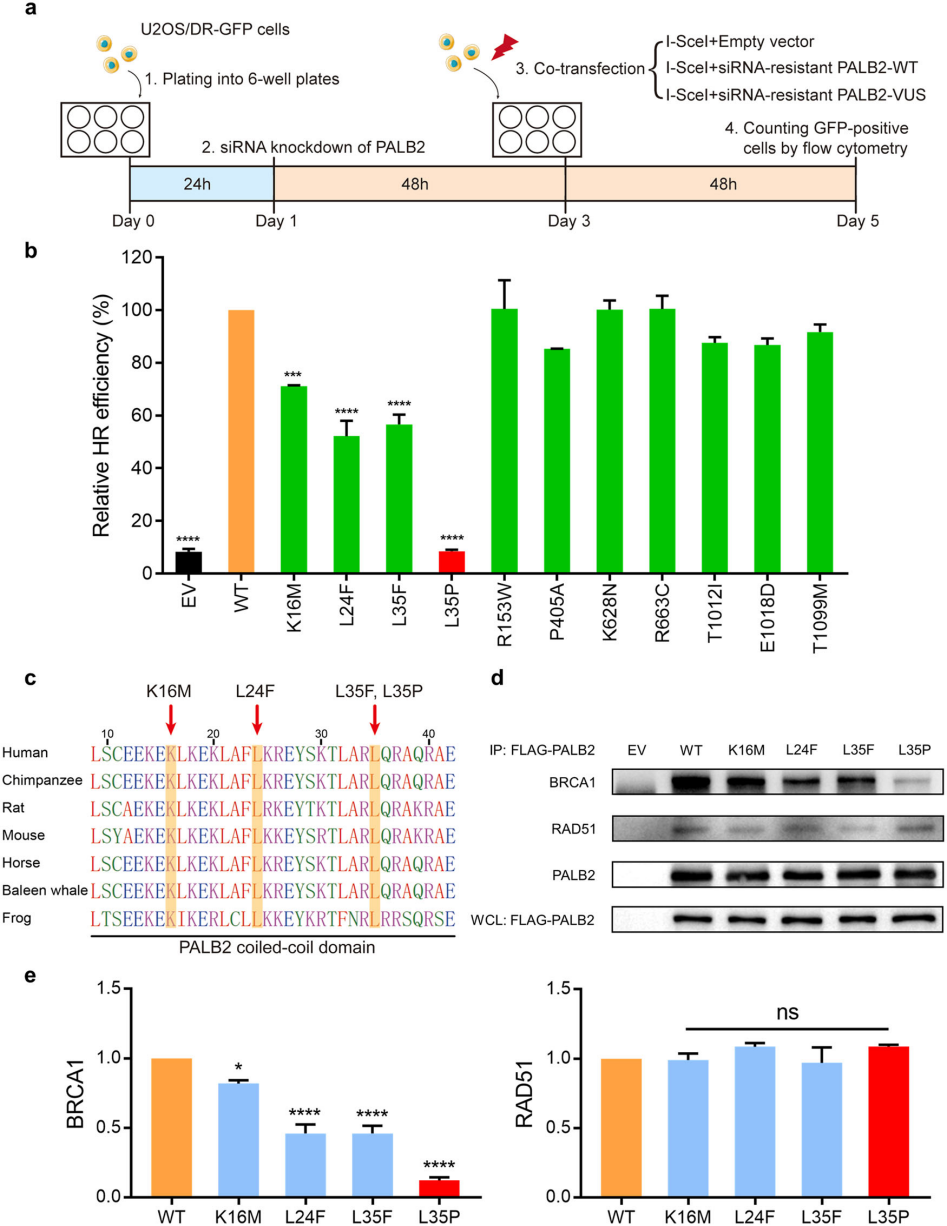
Effects of *PALB2* VUSs on HR activity and BRCA1 interaction. **a** Schematic representation of the HR repair assay used in our study. **b** HR activity of the selected *PALB2* VUSs. PALB2-knockdown U2OS/DR-GFP cells were co-transfected with an I-SceI expression vector and the siRNA-resistant PALB2 constructs (or an empty vector, EV). Data represent the mean percentage ( ± SEM) of GFP-positive cells relative to the WT condition from three independent experiments. Statistical significance was analyzed by one-way ANOVA and Dunnett’s multiple comparisons test. ***P < 0.001; ****P < 0.0001. **c** Amino acid sequence alignment of the PALB2 coiled-coil domain from various species. *PALB2* VUSs that significantly impaired the HR activity were marked on the top. **d** The *PALB2* VUSs disrupted BRCA1-PALB2 interactions. The indicated FLAG-tagged PALB2 constructs (or an empty vector, EV) were transiently transfected in 293 T cells, and PALB2 proteins were immunoprecipitated with anti-FLAG M2 beads. PALB2-binding proteins were analyzed by western blotting. Whole cell lysate (WCL) showed levels of PALB2 expression. **e** Quantification of western blotting band intensities (**d**) from three independent experiments. BRCA1 and RAD51 protein expressions were normalized to PALB2 protein levels and compared to the WT condition. Data presented as mean ± SEM. Statistical significance was analyzed by one-way ANOVA and Dunnett’s multiple comparisons test. *P < 0.05; ****P < 0.0001; ns, not significant.

PALB2 is vital for HR repair in response to DNA DSBs. We first evaluated the HR function of *PALB2* variants using the U2OS/DR-GFP reporter cells. Endogenous PALB2 knockdown efficiency was verified by quantitative real-time PCR and western blotting (Fig. 1b, c). Complemented PALB2 constructs were modified and resistant to siRNA (Fig. 1d). The typical levels of HR activity of PALB2 after siRNA knockdown and siRNA-resistant PALB2 complementation in U2OS/DR-GFP cells were shown in Fig. 1e. Under these settings, we then tested the selected *PALB2* variants (Fig. 2a). As expected, the empty vector (EV) and p.L35P variant completely abrogated the HR activity of PALB2 relative to the wild-type (WT) condition (Fig. 2b). The p.L24F and p.L35F variants showed a considerable impact on the HR activity, retaining only 52.2% (*P* < 0.0001) and 56.7% (*P* < 0.0001) of HR activity respectively. Moreover, the p.K16M also reduced HR activity to 71.2% relative to the WT condition (*P* < 0.001). For the p.R153W, p.P405A, p.K628N, p.R663C, p.T1012I, p.E1018D, and p.T1099M variants, there were no statistical differences in HR activity compared to the WT condition. Interestingly, the amino acid sequence alignment showed that all residues which disrupted PALB2 HR activity were highly conserved in vertebrates (Fig. 2c), suggesting that changes in these amino acid sites may disturb the structure and function of PALB2. Thus, we prioritized the p.K16M, p.L24F, and p.L35F for further functional assessment.

### Effects of *PALB2* variants on BRCA1 interaction

Previous studies have demonstrated that the coiled-coil domain at the N-terminus of PALB2 regulates the BRCA1-PALB2 interaction required for HR at DSBs^11,12^. Since all these selected HR-deficient *PALB2* variants were located in the coiled-coil domain, we next performed co-immunoprecipitation to investigate the impact of these variants on BRCA1 binding. FLAG-tagged PALB2 variants were transiently expressed in 293 T cells and immunoprecipitated with anti-FLAG beads. Quantification of western blotting band intensities suggested that the p.L35P variant almost abrogated the interaction with BRCA1 compared to the WT condition (Fig. 2d, e). The p.L24F and p.L35F variants partially impaired complex formation with BRCA1, both sustaining 46.0% (*P* < 0.0001) of BRCA1 interaction activity. The p.K16M variant presented a slight reduction in BRCA1 binding capacity, retaining 82.0% (*P* < 0.05) of BRCA1 interaction activity compared to the WT condition (Fig. 2d, e). All variants behaved similarly to the WT condition in RAD51 interaction activity (Fig. 2d, e). Taken together, these results suggested that the p.K16M, p.L24F, and p.L35F variants impaired HR function of PALB2 by diminishing their interactions with BRCA1.

### Effects of *PALB2* variants on G2/M checkpoint response

**Fig. 3:**
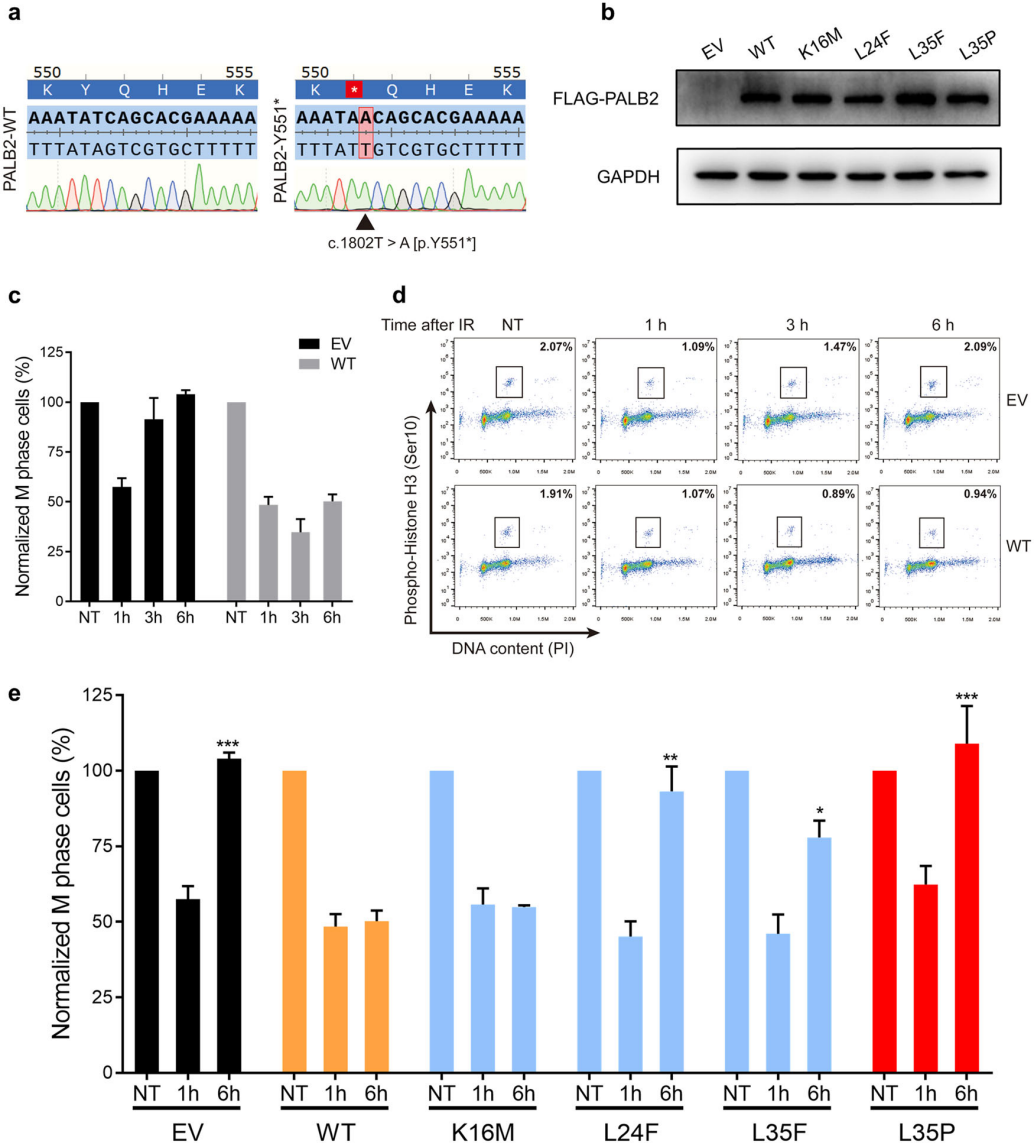
Effects of *PALB2* VUSs on G2/M checkpoint response. **a** Detection of the *PALB2* nonsense variant (c.1802T > A, p.Y551*) of the EUFA1341 cell line. EUFA1341 cells express truncated PALB2 proteins lacking the ability to recruit BRCA2-RAD51. **b** Western blotting analysis of FLAG-tagged PALB2 expression in the EUFA1341 stable cell lines. **c** G2/M checkpoint response in EUFA1341 cells reconstituted with the empty vector (EV) or wild-type (WT) PALB2. Cells were treated with 3 Gy of IR and collected at indicated time points to measure the mitotic index by flow cytometry. Data represent the mean percentage (±SEM) of mitotic cells relative to the untreated cells (NT) from three independent experiments. **d** Representative flow cytometric plots of mitotic cells by phospho-histone H3 (Ser10) and propidium iodide (PI) staining. **e** G2/M checkpoint response in EUFA1341 cells stably expressing PALB2 constructs (or an empty vector, EV). Cells were analyzed before and at indicated time points after 3 Gy of IR. Data represent the mean percentage (±SEM) of mitotic cells relative to the untreated cells (NT) from three independent experiments. Statistical significance was analyzed by one-way ANOVA and Dunnett’s multiple comparisons test. The values of cells expressing indicated constructs were compared to cells expressing the WT PALB2 protein at the same time point. *P < 0.05; **P < 0.01; ***P < 0.001.

Besides HR, PALB2 has been validated as a key component for G2/M checkpoint response^16,30^, and BRCA1-PALB2 interaction is critical for the effective G2/M checkpoint response following DNA damage induced by ionizing radiation (IR)^17^. Therefore, we investigated whether these *PALB2* variants would impair the checkpoint response. To do this, we generated EUFA1341 cell lines stably expressing these *PALB2* variants. EUFA1341 is an FA-N patient-derived skin fibroblast cell line with biallelic mutations in *PALB2*, in which one allele harbors a nonsense mutation and the other allele is deleted (Fig. 3a)^2^. The expression levels of FLAG-PALB2 proteins in EUFA1341 cells were determined by western blotting (Fig. 3b). Following 3 Gy of IR, cells were collected at indicated time points, and mitotic cells were measured by phospho-histone H3 (Ser10) and propidium iodide (PI) staining. Flow cytometric analysis of mitotic cells revealed that the mitotic indexes of both EV and WT PALB2-expressing cells significantly attenuated at 1 h after IR, indicating the potent activation of G2/M checkpoint response in both cell lines (Fig. 3c). Subsequently, the mitotic index of EV-expressing cells gradually increased and fully recovered at 6 h after IR, while cells expressing the WT PALB2 maintained at a low level (Fig. 3c, d). Collectively, these results confirmed the previous findings that PALB2 is a main regulator in the maintenance of the IR-induced G2/M checkpoint response^16,17^.

We further assessed the impacts of these *PALB2* variants on G2/M checkpoint activation and maintenance. As shown in Fig. 3e, all cell lines were able to activate the G2/M checkpoint response at 1 h after 3 Gy of IR. Notably, the G2/M checkpoint maintenance defects were observed for three *PALB2* variants (p.L24F, p.L35F, and p.L35P) at 6 h after IR (Fig. 3e). The p.K16M variant still maintained effective G2/M checkpoint response at 6 h after IR, comparable to cells expressing the WT PALB2.

### *PALB2* variants disrupt PALB2 and RAD51 foci formation

**Fig. 4:**
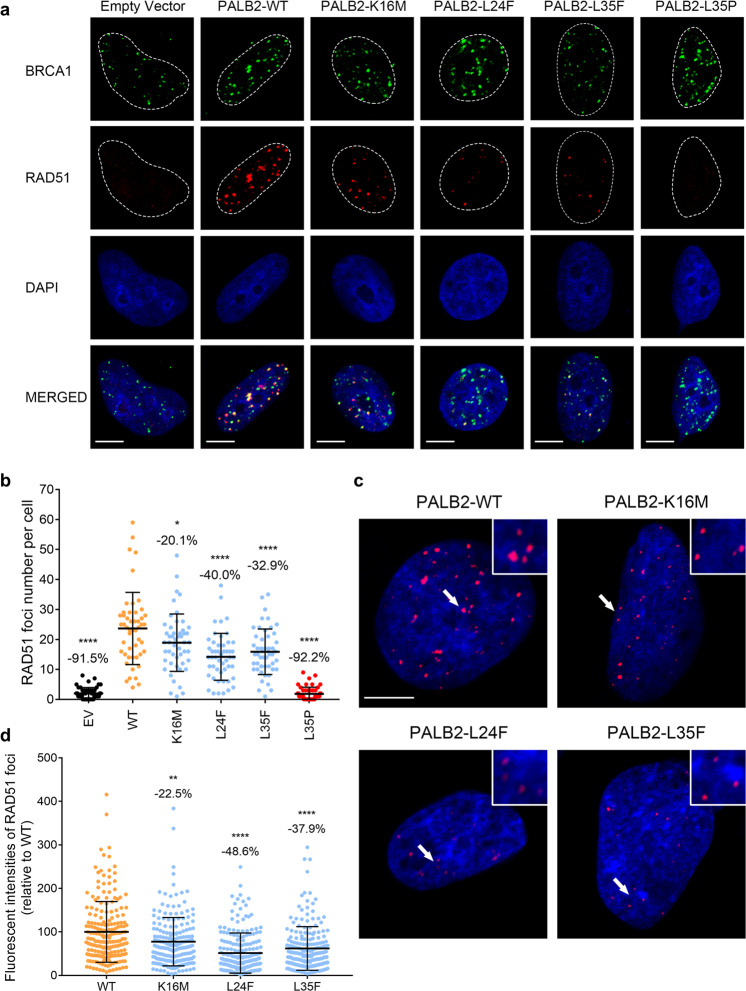
Effects of *PALB2* VUSs on RAD51 foci formation. **a** The *PALB2* VUSs reduced RAD51 foci formation in response to DNA damage. Representative images of RAD51 foci formation in EUFA1341 cell lines stably expressing PALB2 constructs (or an empty vector, EV). BRCA1 was co-stained to determine sites of DNA damage and co-localization with RAD51. Cells were fixed 6 h after 10 Gy of IR and analyzed by immunofluorescence. Scale bar, 5 μm. **b** Quantification of RAD51 foci in BRCA1 foci-positive cells expressing the indicated *PALB2* variant. Results represent the mean values ( ± SD) of three independent experiments (*n* = 50 cells per condition). Statistical significance was analyzed by one-way ANOVA and Dunnett’s multiple comparisons test. *P < 0.05; ****P < 0.0001. **c** The *PALB2* VUSs diminished the RAD51 foci intensity in response to DNA damage. Representative immunofluorescence images of RAD51 foci intensity in BRCA1 foci-positive EUFA1341 cells expressing the indicated *PALB2* variants. Scale bar, 5 μm. **d** Quantification of fluorescent intensities of RAD51 foci in BRCA1 foci-positive cells expressing the indicated *PALB2* variants. Results represent the mean values ( ± SD) of 200 RAD51 foci (relative to the WT mean). Statistical significance was analyzed by Kruskal-Wallis test and Dunn’s multiple comparisons test. **P < 0.01; ****P < 0.0001.

Mechanistically, the BRCA1-PALB2-BRCA2 complex recruits RAD51 and stimulates the formation of RAD51 nucleofilaments, which is critical for HR^11,12^. Thus, we subsequently assessed the HR competency of *PALB2* variants using IR-induced PALB2 and RAD51 foci formation. Following exposure to 10 Gy of IR, EUFA1341 cells expressing the different variants were subjected to immunofluorescence staining for PALB2 and RAD51 foci. As shown in Supplementary Fig. 1, the p.L35P variant failed to form any PALB2 foci. The p.L24F and p.L35F variants showed partially impaired PALB2 foci formation compared to the WT condition, while the p.K16M variant presented a more minor effect. Consistently, cells expressing the p.L35P variant showed a substantial reduction of over 90% in RAD51 foci formation, similar to the EV condition (Fig. 4a, b). The p.L24F and p.L35F variants caused moderate decreases in the mean number of RAD51 foci, presenting a reduction of 40.0% and 32.9%, respectively. Cells expressing the p.K16M variant only showed a slight decrease in RAD51 foci formation relative to the WT condition. We further evaluated the fluorescent intensities of RAD51 foci of these *PALB2* variants. The p.L24F and p.L35F variants presented partially attenuated RAD51 foci intensity compared to the WT condition, while the p.K16M variant displayed a mild effect on RAD51 foci intensity (Fig. 4c, d).

### Cellular sensitivity to DNA damaging agents

**Fig. 5:**
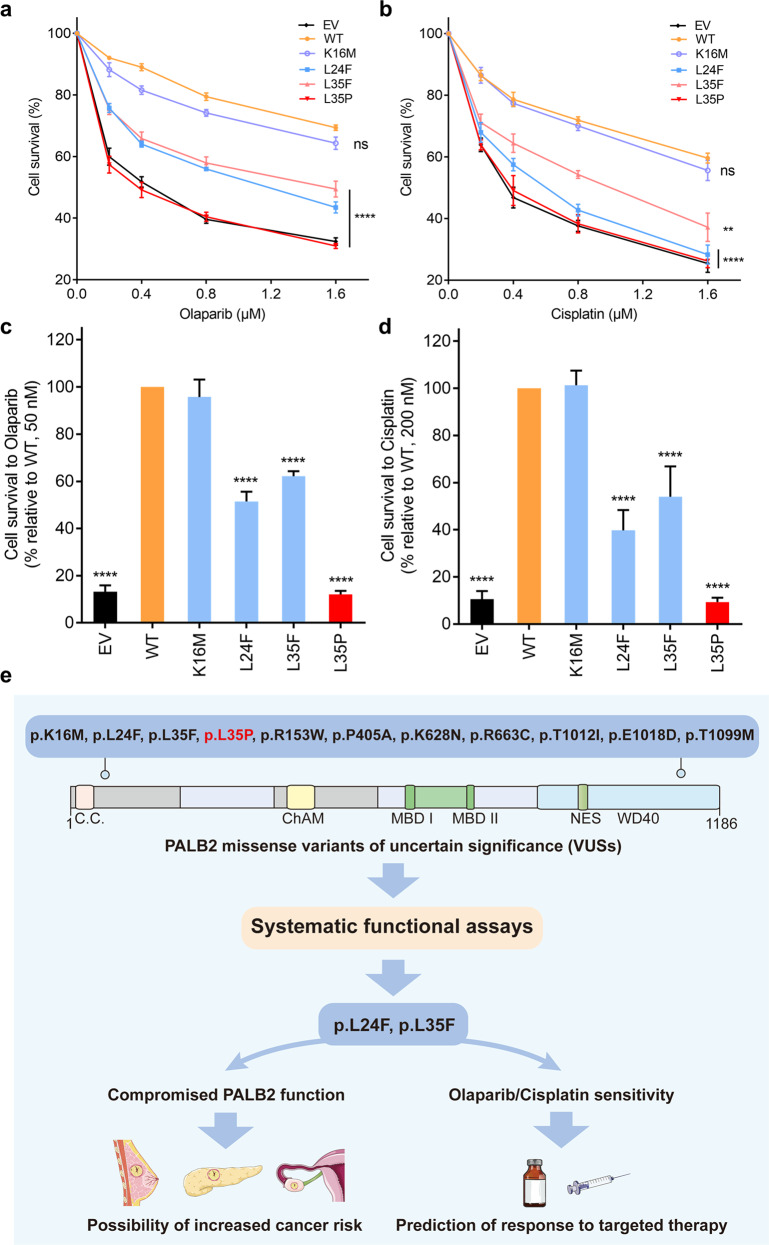
Effects of *PALB2* VUSs on cellular sensitivity to PARP inhibitor and cisplatin. **a**, **b** Proliferation-based survival of EUFA1341 stable cell lines after exposure to incremental doses of PARP inhibitor (olaparib) or cisplatin. Cells were incubated with drugs for 96 h. Data represent the mean percentage (± SEM) of viability relative to untreated cells from three independent experiments. Statistical significance was analyzed by one-way ANOVA and Dunnett’s multiple comparisons test. **P < 0.01; ****P < 0.0001; ns Not significant. **c**, **d** Clonogenic survivals of EUFA1341 stable cell lines after exposure to olaparib or cisplatin. Cells were exposed to the indicated concentrations of olaparib (50 nM) or cisplatin (200 nM) for 10 days, after which surviving colonies were counted. Data represent the mean percentage ( ± SEM) of survival relative to the WT condition from three independent experiments. Statistical significance was analyzed by one-way ANOVA and Dunnett’s multiple comparisons test. ****P < 0.0001. **e** Schematic summary of the present findings.

Since HR deficiency is associated with cellular sensitivity to PARPi and platinum agents^22^, we performed a cellular proliferation assay to evaluate the effects of the *PALB2* variants on the PARPi (olaparib) and cisplatin sensitivity. Consistent with previous findings^22^, EUFA1341 cells expressing the p.L35P variant showed striking vulnerability to olaparib and cisplatin treatment comparable to the EV condition. We also observed that the p.L24F and p.L35F variants displayed marked hypersensitivity to olaparib and cisplatin than the WT control. Unexpectedly, despite presenting reduced HR activity and RAD51 foci formation, the p.K16M variant behaved similarly to the WT control with regard to olaparib and cisplatin sensitivity (Fig. 5a, b). We further investigated the influence of the *PALB2* variants on the olaparib and cisplatin sensitivity using a clonogenic survival assay. Cells were exposed to prolonged treatments with lower doses of olaparib and cisplatin. Consistently, the p.L24F, p.L35F, and p.L35P variants conferred greater sensitivity to olaparib and cisplatin than the WT control, whereas the p.K16M variant showed a WT level of resistance to olaparib and cisplatin (Fig. 5c, d). Consequently, the *PALB2* variants that severely impaired HR function may serve as therapeutic targets for both PARPi and platinum agents.

## Discussion

In this study, we assessed the influence of *PALB2* missense variants on protein function using systematic functional assays combined with in silico predictions. These results provided a preliminary interpretation of the pathogenicity of *PALB2* VUSs identified in the Chinese breast cancer population (Fig. 5e). Out of the 10 *PALB2* missense variants evaluated in this study, we identified two variants, p.L24F and p.L35F, that disrupted the HR function of PALB2. Consistently, these two variants partially impaired BRCA1-PALB2 interactions and reduced RAD51 foci formation in response to DNA damage. Moreover, the p.L24F and p.L35F variants abrogated IR-induced G2/M checkpoint maintenance and conferred increased sensitivity to olaparib and cisplatin.

The variants that impaired PALB2 function are located in the coiled-coil domain of PALB2, which is consistent with previous findings indicating that the coiled-coil domain is a hotspot for *PALB2* loss-of-function variants^23–25^. Mechanistically, we found that the p.L24F and p.L35F variants diminished BRCA1-PALB2 interactions, which was in line with their compromised HR activity and reduced RAD51 foci formation. Thus, these results highlighted the importance of the BRCA1-PALB2 interaction in HR activity as well as RAD51 recruitment. These variants with impact on PALB2 function may be related to the increased risk of breast cancer. Of note, variants in the coiled-coil domain may also affect the homodimerization of PALB2, which is mediated by an antiparallel coiled-coil structure^31–33^. Variant of the key residue L24 markedly reduces the PALB2 homodimer stability and attenuates PALB2 activity in DNA damage repair^33^. Moreover, the PALB2 N-terminus, including the coiled-coil domain, is the RAD51-binding region that enhances RAD51-mediated strand exchange^14,15^. Hence, further explorations are needed to elucidate the multifaceted effects of these variants on PALB2 function. Interestingly, different amino acid substitutions at the same site may cause diverse effects on PALB2 function. This is supported by our results indicating that the p.L35P variant completely abrogated the HR function of PALB2 while the p.L35F variant showed only partially compromised PALB2 function. We surmise that replacing the Leu35 residue with Pro may cause a broader disruption of the coiled-coil domain.

In response to DNA damage, normal cells activate cell cycle checkpoints to arrest cell cycle progression, and effective checkpoint maintenance is essential for DNA damage repair and genome stability^34^. Our results identified two *PALB2* missense variants (p.L24F and p.L35F), along with the previously reported p.L35P variant, that abrogated IR-induced G2/M checkpoint maintenance. Notably, the p.L24F and p.L35F variants not only reduced HR activity of PALB2 but also abrogated G2/M checkpoint maintenance, which may exacerbate genomic instability and lead to cancer susceptibility.

PARPi olaparib has been recently approved for the treatment of human epidermal growth factor receptor 2 (HER2)-negative metastatic breast cancer in germline *BRCA1/2*-mutated patients^35^. BRCA1/2-deficient tumors are unable to accurately repair the DNA DSBs induced by PARPi via the HR pathway, resulting in cell death, known as synthetic lethality^36^. Notably, PALB2 is also vital for HR-mediated DNA repair, and PALB2 deficiency in cells confers impaired HR function. Recently, several studies have demonstrated benefits of PARPi treatment in breast cancer patients with germline *PALB2* variants^37,38^. Tung et al. reported that the response rate and progression-free survival with olaparib treatment for metastatic breast cancer patients with germline *PALB2* variants were 82% and 13.3 months, expanding the population of patients with metastatic breast cancer who may derive benefit from PARPi^38^. In the present study, our analysis identified two missense variants (p.L24F and p.L35F) that presented increased sensitivity to olaparib and cisplatin, suggesting that PARPi and platinum agents treatment may be effective strategies for patients with breast cancer carrying these two variants. Interestingly, although the p.K16M variant showed impaired HR function, it did not confer sensitivity to olaparib and cisplatin, indicating that the residual HR competency of p.K16M is sufficient to ensure a WT-like profile in drug sensitivity. Thus, further assessments are required to better define the threshold of HR impairment that may confer sensitivity to targeted therapies.

According to National Comprehensive Cancer Network (NCCN) guidelines, women with *PALB2* pathogenic or likely pathogenic variants are recommended to perform an annual mammogram with consideration of tomosynthesis and breast magnetic resonance imaging starting at 30 years of age for early detection of tumors^39^. However, a major challenge for genetic risk assessment is to quantify the relationship between PALB2 function deficiency and increased breast cancer risk. For instance, most of the likely pathogenic variants we identified in this study partially impaired PALB2 function. It is unknown whether this extent of functional defects translates into increased cancer risk. As such, caution is required when assessing the cancer risk of individuals with these *PALB2* variants. With the collection of population-based data combined with systematic functional verification, the clinical classification of *PALB2* VUSs will be more accurate. Of note, the overlap between in silico predictions and functional analysis is low. The 10 *PALB2* missense variants selected in our research were predicted to be pathogenic by at least two in silico algorithms, while only 2 of these variants were verified to compromise PALB2 function. The discordance was mainly due to the SIFT and Polyphen-2 predictions, suggesting that in silico prediction for deleterious variants may result in false positives.

Taken together, our findings can be integrated into population-based data for accurate classification of *PALB2* VUSs and advancing individualized treatment regimens for better clinical outcomes.

## Methods

### Cell culture

U2OS/DR-GFP HR reporter cells were kindly gifted by the laboratories of Dr. Bing Xia and Dr. Jun Huang^22^. EUFA1341 cells were kindly gifted by Dr. Martin A Rooimans^2^. These cells and 293 T cells (Cell Bank of the Chinese Academy of Sciences, Shanghai, China) were cultured in DMEM supplemented with 10% fetal bovine serum and 1% penicillin/streptomycin solution. All cell lines were grown at 37 °C with 5% CO_2_. All cell lines were certified to be mycoplasma-free using a mycoplasma detection kit (Yeasen, Shanghai, China).

### Constructs and lentiviral infection

The pOZ-FH-C1-PALB2 vector was kindly gifted by the laboratories of Dr. Bing Xia and Dr. Jun Huang. *PALB2* variants were introduced into the pOZ-FH-C1-PALB2 vector using a site-directed mutagenesis kit (Yeasen, Shanghai, China). Constructs were verified by Sanger sequencing. For stable expression of PALB2 in the EUFA1341 cells, FLAG-tagged PALB2 variants were cloned into the pCDH-CMV-MCS-EF1α-Puro lentiviral vector (System Biosciences, Palo Alto, CA, USA) using EcoRI/BamHI restriction sites. Following lentiviral packaging, EUFA1341 cells were infected with lentivirus to generate stable cell lines expressing PALB2 variants.

### Quantitative real-time PCR

Total RNA was extracted using TRIzol reagent (Invitrogen, Carlsbad, CA, USA), and cDNA was synthesized with the cDNA synthesis kit (Yeasen, Shanghai, China). Real-time PCR was carried out using the SYBR Green kit (Yeasen, Shanghai, China) on the Applied Biosystems 7500 Fast Real-Time PCR System. Relative mRNA expression was determined using the 2^-ΔΔCt^ method. The following primers were used: PALB2 (Human)-forward: GTCAGTGACCCTAGTGGTGAG; PALB2 (Human)-reverse: CAATCTGAGTGAATCAGTGCCAA; GAPDH (Human)-forward: ACAACTTTGGTATCGTGGAAGG; GAPDH (Human)-reverse: GCCATCACGCCACAGTTTC.

### Homologous recombination assay

The HR repair assay was carried out using U2OS/DR-GFP reporter cells^40^. Cells were first transfected with a PALB2 siRNA to deplete endogenous PALB2 using the Lipofectamine RNAiMAX reagent (Invitrogen, Carlsbad, CA, USA). After 48 h of transfection, 500,000 cells were collected for each condition and then co-transfected with 3 μg of I-SceI expression vector and 1.5 μg of various siRNA-resistant pOZ-FH-C1-PALB2 constructs (or pOZC empty vector) using Gene Pulser Xcell (Bio-Rad, Hercules, CA, USA). After 48 h of the second transfection, cells were collected, and GFP-positive cells were quantified by flow cytometry (Beckman Coulter, Brea, CA, USA). The PALB2 siRNA sequence was 5’-UCAUUUGGAUGUCAAGAAAdTdT-3’ and the control sequence was 5’-UUCGAACGUGUCACGUCAAdTdT-3’.

### Immunoprecipitation (IP) and western blotting

The pOZ-FH-C1-PALB2 constructs were transfected into 293 T cells using the Liposomal transfection reagent (Yeasen, Shanghai, China). After 48 h of transfection, cells were lysed with IP Lysis Buffer (Thermo Fisher Scientific, Waltham, MA, USA) supplemented with protease inhibitors (Thermo Fisher Scientific, Waltham, MA, USA). The FLAG-tagged PALB2 were IPed with anti-FLAG M2 magnetic beads (Sigma, St. Louis, MO, USA) overnight. For western blotting analyses, protein lysates were separated by 8–10% SDS-PAGE, transferred to PVDF membranes and probed with relevant antibodies, followed by ECL detection. The antibodies used were FLAG (Sigma Cat# F1804, 1:1000, St. Louis, MO, USA), PALB2 (Absin Cat# abs120051, 1:500, Shanghai, China), BRCA1 (Millipore Cat# 07-434, 1:5000, Burlington, MA, USA), RAD51 (Abcam Cat# ab133534, 1:5000, Cambridge, UK), and GAPDH (Boster Cat# BM1985, 1:2000, Pleasanton, CA, USA). Relative protein expression was determined using ImageJ.

### G2/M checkpoint assay

EUFA1341 stable cells were seeded in 6-well plates at 5 × 10^5^ cells per well. The next day, cells were exposed to 3 Gy of IR and incubated for indicated time periods before collection. Collected cells were fixed in 70% ice-cold ethanol overnight. Fixed cells were permeabilized with 0.25% Triton X-100 in phosphate-buffered saline (PBS) on ice for 15 min and then stained with Alexa Fluor 488-conjugated phospho-histone H3 (Ser10) Ab (Cell Signaling Technology Cat#3465, 1:50, Danvers, MA, USA) in PBS containing 1% bovine serum albumin (BSA) for 2 h. Before analysis, propidium iodide (PI)/RNase staining buffer (BD Biosciences Cat#550825, San Diego, CA, USA) was added to stain DNA. M-phase cells were quantified by flow cytometry (Beckman Coulter, Brea, CA, USA).

### Immunofluorescence

EUFA1341 stable cells were seeded on glass-bottom dishes at 100,000 cells per dish. The next day, cells were irradiated with 10 Gy and processed for immunofluorescence after 6 h of recovery. Cells were washed with PBS and fixed in 4% paraformaldehyde for 15 min at room temperature. Following PBS washing, cells were permeabilized with 0.1% Triton X-100 for 5 min and then incubated in blocking solution containing 1% BSA for 1 h. Cells were then incubated with primary antibodies anti-BRCA1 (Santa Cruz Biotechnology Cat# sc-6954, 1:100, Dallas, TX, USA), anti-RAD51 (Abcam Cat# ab133534, 1:1000, Cambridge, UK), anti-FLAG (Sigma Cat# F1804, 1:1000, St. Louis, MO, USA), and anti-γH2AX (Abcam Cat# ab81299, 1:250, Cambridge, UK) overnight at 4 °C. After PBS washing, cells were incubated with second antibodies Alexa Fluor 488 goat anti-mouse (Abcam Cat# ab150113, 1:500, Cambridge, UK) and Alexa Fluor 647 goat anti-rabbit (Abcam Cat# ab150079, 1:500, Cambridge, UK) for 1 h at room temperature. Nuclei were stained for 10 min with 4′,6-diamidino-2-phenylindole (DAPI) before analysis. Images were captured using a ZEISS LSM 710 microscope, and the ZEN 3.3 (blue edition) software (ZEISS, Oberkochen, GER) was used for RAD51 foci analysis.

### Drug sensitivity assay

For proliferation-based olaparib and cisplatin sensitivity assays, EUFA1341 stable cells were seeded at 1500 cells per well of a 96-well plate. The next day, cells were treated with the indicated doses of olaparib (Selleck, Cat# S1060, Houston, TX, USA) or cisplatin (Selleck, Cat# S1166, Houston, TX, USA). Cells were incubated with drugs for 96 h and cell viability was measured using the CCK-8 kit (Dojindo, Kumamoto, JPN) according to manufacturer’s instructions. For the clonogenic drug sensitivity assay, EUFA1341 stable cells were seeded in six-well plates at two different densities: 1000 cells per well for PALB2 VUSs and 3000 cells per well for the empty vector and the p.L35P variant. Cells were incubated with the indicated doses of olaparib or cisplatin and allowed to form colonies for 10 days. Colonies were fixed, stained with crystal violet, and counted.

### Statistical analysis

Data were analyzed in GraphPad Prism 7.0 (GraphPad Software, San Diego, CA, USA). Statistical significance was determined using the Student’s *t*-test, one-way ANOVA, or Kruskal-Wallis test. *P*-values < 0.05 were considered statistically significant.

## Supplementary information

Supplementary information

## Data Availability

All data generated or analyzed during this study are included in this article. All the uncropped western blots generated during this study are available in Supplementary Fig. 2.

## Code availability

All data generated or analyzed were described in the “Methods” section. No custom codes were used.

## References

[1] Xia B (2006). Control of BRCA2 cellular and clinical functions by a nuclear partner, PALB2. Mol. Cell.

[2] Xia B (2007). Fanconi anemia is associated with a defect in the BRCA2 partner PALB2. Nat. Genet..

[3] Reid S (2007). Biallelic mutations in PALB2 cause Fanconi anemia subtype FA-N and predispose to childhood cancer. Nat. Genet..

[4] Yang X (2020). Cancer Risks Associated With Germline PALB2 Pathogenic Variants: An International Study of 524 Families. J. Clin. Oncol..

[5] Antoniou AC (2014). Breast-cancer risk in families with mutations in PALB2. N. Engl. J. Med..

[6] Nepomuceno TC (2021). PALB2 Variants: Protein domains and cancer susceptibility. Trends Cancer.

[7] Dorling L (2021). Breast cancer risk genes - Association analysis in more than 113,000 women. N. Engl. J. Med..

[8] Heikkinen T (2009). The breast cancer susceptibility mutation PALB2 1592delT is associated with an aggressive tumor phenotype. Clin. Cancer Res..

[9] Cybulski C (2015). Clinical outcomes in women with breast cancer and a PALB2 mutation: A prospective cohort analysis. Lancet Oncol..

[10] Ducy M (2019). The tumor suppressor PALB2: Inside Out. Trends Biochem. Sci..

[11] Zhang F, Fan Q, Ren K, Andreassen PR (2009). PALB2 functionally connects the breast cancer susceptibility proteins BRCA1 and BRCA2. Mol. Cancer Res..

[12] Sy SM, Huen MS, Chen J (2009). PALB2 is an integral component of the BRCA complex required for homologous recombination repair. Proc. Natl. Acad. Sci. USA..

[13] Park JY (2014). Breast cancer-associated missense mutants of the PALB2 WD40 domain, which directly binds RAD51C, RAD51 and BRCA2, disrupt DNA repair. Oncogene.

[14] Buisson R (2010). Cooperation of breast cancer proteins PALB2 and piccolo BRCA2 in stimulating homologous recombination. Nat. Struct. Mol. Biol..

[15] Dray E (2010). Enhancement of RAD51 recombinase activity by the tumor suppressor PALB2. Nat. Struct. Mol. Biol..

[16] Menzel T (2011). A genetic screen identifies BRCA2 and PALB2 as key regulators of G2 checkpoint maintenance. EMBO Rep..

[17] Simhadri S (2019). PALB2 connects BRCA1 and BRCA2 in the G2/M checkpoint response. Oncogene.

[18] Ma J (2012). PALB2 interacts with KEAP1 to promote NRF2 nuclear accumulation and function. Mol. Cell Biol..

[19] Bleuyard JY (2017). MRG15-mediated tethering of PALB2 to unperturbed chromatin protects active genes from genotoxic stress. Proc. Natl. Acad. Sci. USA..

[20] Murphy AK (2014). Phosphorylated RPA recruits PALB2 to stalled DNA replication forks to facilitate fork recovery. J. Cell Biol..

[21] Buisson R (2014). Breast cancer proteins PALB2 and BRCA2 stimulate polymerase eta in recombination-associated DNA synthesis at blocked replication forks. Cell Rep..

[22] Foo TK (2017). Compromised BRCA1-PALB2 interaction is associated with breast cancer risk. Oncogene.

[23] Wiltshire T (2020). Functional characterization of 84 PALB2 variants of uncertain significance. Genet. Med..

[24] Rodrigue A (2019). A global functional analysis of missense mutations reveals two major hotspots in the PALB2 tumor suppressor. Nucleic Acids Res..

[25] Boonen R (2019). Functional analysis of genetic variants in the high-risk breast cancer susceptibility gene PALB2. Nat. Commun..

[26] Zhang K (2017). Germline mutations of PALB2 gene in a sequential series of Chinese patients with breast cancer. Breast Cancer Res. Treat..

[27] Sim NL (2012). SIFT web server: predicting effects of amino acid substitutions on proteins. Nucleic Acids Res..

[28] Tavtigian SV (2006). Comprehensive statistical study of 452 BRCA1 missense substitutions with classification of eight recurrent substitutions as neutral. J. Med. Genet..

[29] Adzhubei IA (2010). A method and server for predicting damaging missense mutations. Nat. Methods.

[30] Cotta-Ramusino C (2011). A DNA damage response screen identifies RHINO, a 9-1-1 and TopBP1 interacting protein required for ATR signaling. Science.

[31] Sy SM, Huen MS, Zhu Y, Chen J (2009). PALB2 regulates recombinational repair through chromatin association and oligomerization. J. Biol. Chem..

[32] Buisson R, Masson JY (2012). PALB2 self-interaction controls homologous recombination. Nucleic Acids Res..

[33] Song F (2018). Antiparallel Coiled-Coil Interactions Mediate the Homodimerization of the DNA Damage-Repair Protein PALB2. Biochemistry.

[34] Löbrich M, Jeggo PA (2007). The impact of a negligent G2/M checkpoint on genomic instability and cancer induction. Nat. Rev. Cancer.

[35] Robson M (2017). Olaparib for metastatic breast cancer in patients with a germline BRCA mutation. N. Engl. J. Med..

[36] Chan CY, Tan KV, Cornelissen B (2021). PARP Inhibitors in Cancer Diagnosis and Therapy. Clin. Cancer Res..

[37] Grellety T (2020). Dramatic response to PARP inhibition in a PALB2-mutated breast cancer: moving beyond BRCA. Ann. Oncol..

[38] Tung NM (2020). TBCRC 048: Phase II Study of Olaparib for Metastatic Breast Cancer and Mutations in Homologous Recombination-Related Genes. J. Clin. Oncol..

[39] Daly MB (2021). Genetic/Familial high-risk assessment: breast, ovarian, and pancreatic, Version 2.2021, NCCN clinical practice guidelines in oncology. J. Natl. Compr. Canc. Netw..

[40] Simhadri S (2014). Male fertility defect associated with disrupted BRCA1-PALB2 interaction in mice. J. Biol. Chem..

