# Supplementary material for: Functional assessment of missense variants of uncertain significance in the cancer susceptibility gene PALB2
Source: NPJ Breast Cancer. 2022 Jul 19;8:86. doi: 10.1038/s41523-022-00454-6 (PMC9296472; doi:10.1038/s41523-022-00454-6)

Supplementary Figures

Supplementary figure 1

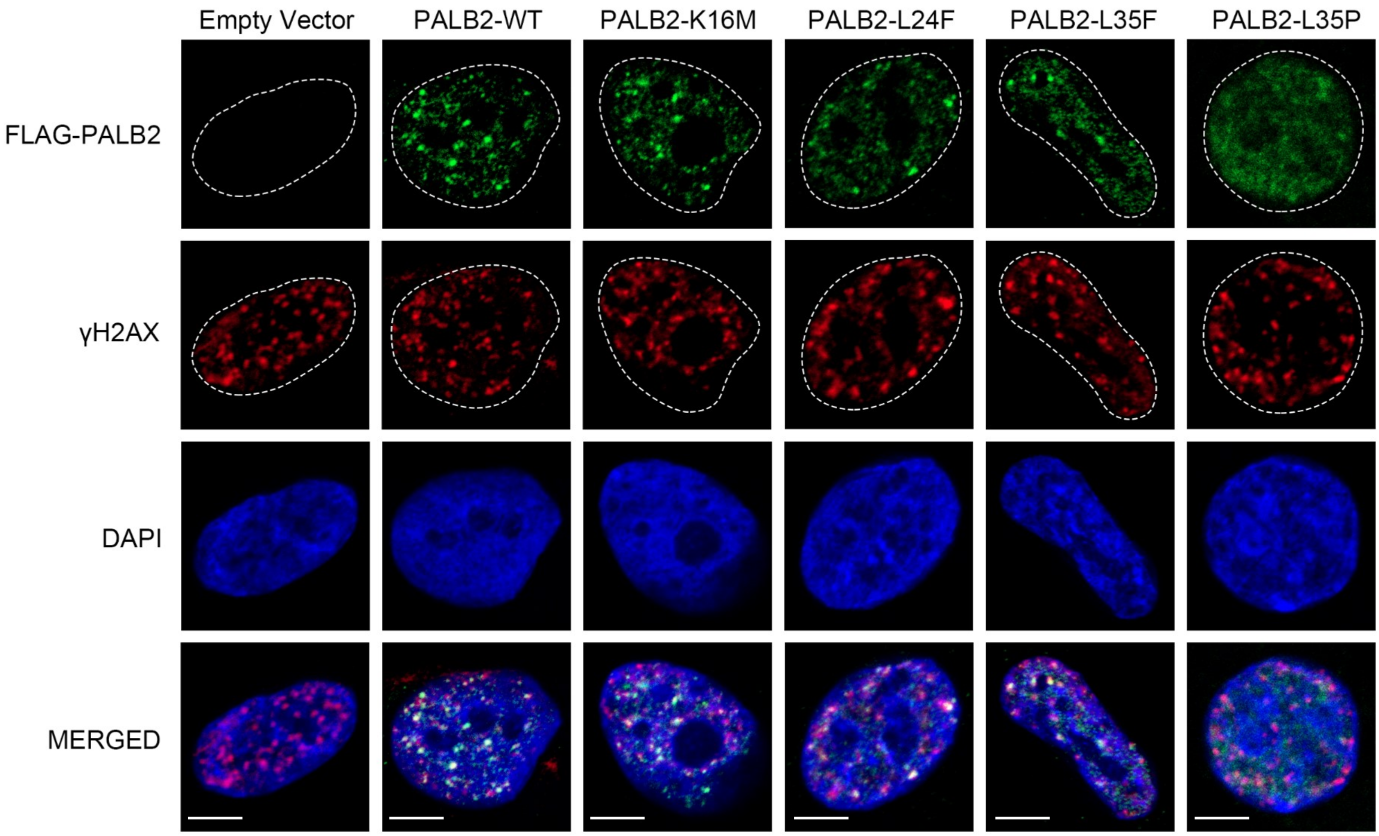

Supplementary Fig. 1 Effects of *PALB2* VUSs on PALB2 foci formation. Representative images of PALB2 foci formation in EUFA1341 cell lines stably expressing PALB2 constructs (or an empty vector, EV). Phosphorylated histone H2AX (γH2AX) was co-stained to determine sites of DNA damage and co-localization with PALB2. Cells were fixed 6 hours after 10 Gy of IR and analyzed by immunofluorescence. Scale bar, 5 μm.

Supplementary figure 2: Uncropped western blot images

Figure 1C

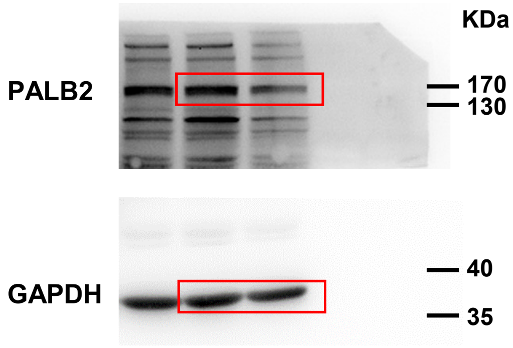

Figure 2D

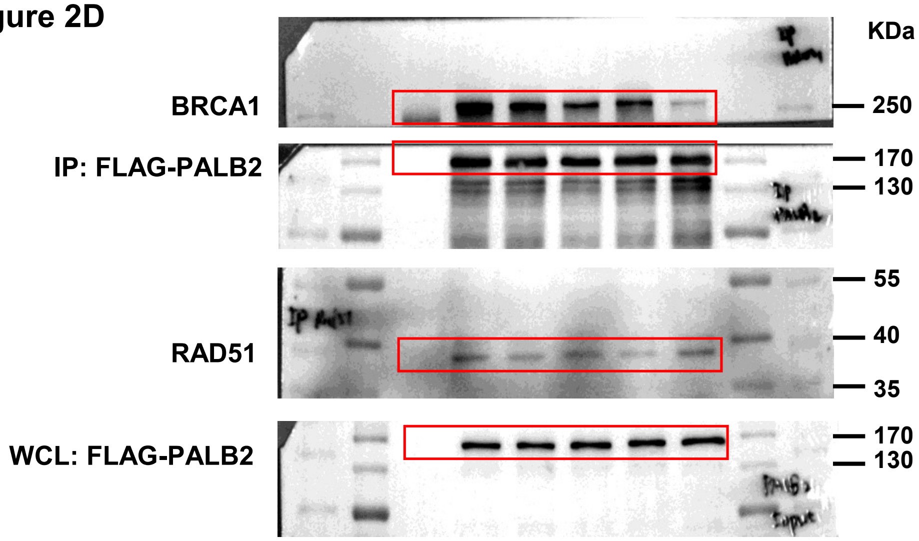

Figure 3B

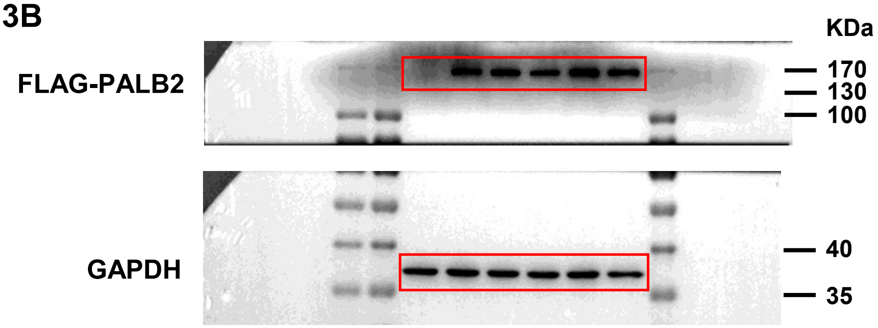

Supplement: Supplementary file 1 — Supplementary information [file 41523_2022_454_MOESM1_ESM.pdf]
